# Autism Spectrum Disorder and Prenatal or Early Life Exposure to Pesticides: A Short Review

**DOI:** 10.3390/ijerph182010991

**Published:** 2021-10-19

**Authors:** Alessandro Miani, Giovanni Imbriani, Giovanni De Filippis, Donato De Giorgi, Luigi Peccarisi, Manuela Colangelo, Manuela Pulimeno, Maria Domenica Castellone, Giuseppe Nicolardi, Giancarlo Logroscino, Prisco Piscitelli

**Affiliations:** 1 Italian Society of Environmental Medicine (SIMA), 20123 Milan, Italy; alessandro.miani@gmail.com; 2 Department of Environmental Science and Policy, University of Milan, 20133 Milan, Italy; 3 Department of Biological and Environmental Sciences and Technologies, Università del Salento, 73100 Lecce, Italy; giovanni.imbriani@unisalento.it (G.I.); giuseppe.nicolardi@unisalento.it (G.N.); 4 Local Health Authority ASL Le, 73100 Lecce, Italy; giov.defilippis@gmail.com (G.D.F.); donatodg@libero.it (D.D.G.); ginopeccarisi@libero.it (L.P.); 5 Medical Professional Association (OMCEO), 73100 Lecce, Italy; 6 Italian Association for Health, Environment and Society (AISAS), 73023 Lizzanello, Italy; Manuela.colangelo95@gmail.com; 7 Staff UNESCO Chair on Health Education and Sustainable Development, Federico II University, 80100 Naples, Italy; pulimeno.m@tiscali.it; 8 National Research Council (CNR-IEOS), 80131 Naples, Italy; mcastell@unina.it; 9 Department of Basic Medicine Sciences, Neuroscience, and Sense Organs, University of Bari “Aldo Moro”, 70121 Bari, Italy; giancarlo.logroscino@uniba.it

**Keywords:** autism spectrum disorder, pesticides, pregnancy, prenatal and postnatal exposure, children

## Abstract

*Background*: Autism spectrum disorder (ASD) diagnoses have rapidly increased globally. Both environmental and genetic factors appear to contribute to the development of ASD. Several studies have shown a potential association between prenatal or postnatal pesticide exposure and the risk of developing ASD. *Methods*: We reviewed the available literature concerning the relationship between early life exposure to pesticides used in agriculture, such as organochlorines, organophosphates and pyrethroids, and ASD onset in childhood. We searched on Medline and Scopus for cohort or case–control studies published in English from 1977 to 2020. *Results*: A total of seven articles were selected for the review. We found a remarkable association between the maternal exposure to pyrethroid, as well as the exposure to organophosphate during pregnancy or in the first years of childhood, and the risk of ASD onset. This association was found to be less evident with organochlorine pesticides. Pregnancy seems to be the time when pesticide exposure appears to have the greatest impact on the onset of ASD in children. *Conclusions*: Among the different environmental pollutants, pesticides should be considered as emerging risk factors for ASD. The potential association identified between the exposure to pesticides and ASD needs to be implemented and confirmed by further epidemiological studies based on individual assessment both in outdoor and indoor conditions, including multiple confounding factors, and using statistical models that take into account single and multiple pesticide residues.

## 1. Introduction

Autism spectrum disorder (ASD) refers to a set of neurodevelopmental conditions characterized by early difficulties in social communication and unusually limited and repetitive behaviors and interests [1].

Over the past few decades, the diagnosis of ASD has rapidly increased globally. According to the Autism and Developmental Disabilities Monitoring Network (ADDM), in a group of programs funded by the American Centers for Disease Control and Prevention (CDC) used to estimate the number of children with autism spectrum disorder (ASD) and other developmental disabilities across the United States from 2000 to 2016, the prevalence of ASD has grown exponentially since 2000, increasing from 1 in 150 newborns in the 8-year-old pediatric population to 1 out of 54 cases in the pediatric population aged 8 years in 2016 [2,3,4].

A study reported by the World Health Organization, reviewing the results of several studies, indicated an overall prevalence of ASD involving 1 out of 160 children [5].

In general, what has been observed from epidemiological studies conducted over the last 50 years seems to show a steady increase in prevalence of this condition. Among the reasons for this expansion are several factors such as the use of better diagnostic methods, the extension of diagnostic criteria and greater attentiveness [6]. However, even these factors could not entirely provide an explanation for the rapid increase observed in the incidence of ASD [7,8].

The genetic component of autism spectrum disorder has been the subject of numerous studies that have led to the identification of mutations of various genes involved in the development of the nervous system [9,10,11,12], which could lead to a transient disruption of brain developmental sequence [13]. The environmental factors framed from an epigenetic perspective, emerged as further potential contributors to the etiology of autism spectrum disorder, thus highlighting the need for a deeper understanding of gene–environment interactions in the pathophysiology of ASD [14].

In addition to environmental pollutants originating from air pollution such as Particulate Matter (PM), NO_2_, SO_2_ and O_3_, which have been extensively studied and correlated with a greater risk of ASD onset, the pesticides used in agriculture have been suggested to play an important role in the etiology of autism spectrum disorder. Pesticides are widely used in agriculture to kill pests that damage crops or other vegetal products, but also for public health purposes to destroy vectors of infectious diseases (i.e., mosquitoes carrying plasmodium malariae, etc.). With the growth of agricultural production, the number and quantity of pesticides for plant and crop protection has also grown, leading to environmental contamination and risks to human health worldwide [15,16]. The global use of pesticides is estimated to be over 2.27 billion kg each year in agricultural, residential, commercial or industrial settings [17]. By their nature, pesticides are potentially toxic to other organisms, including humans, and need to be used safely and properly disposed of. In many countries, farmers need to follow a compulsory training to obtain a specific permission to use pesticides.

Several experimental and epidemiological studies have highlighted the association between neurodevelopmental disorders and pre/postnatal or chronic exposure to pesticides. In addition, a recent systematic review of human and preclinical models has suggested that the gestational exposure to certain organophosphate agents could be linked to the clinical signs of ASD [18]. The aim of this review of studies carried out up until 2020 is to perform a descriptive analysis of any association that has been found between the use of pesticides (to which most of the world’s population is exposed) and autism spectrum disorder, summarizing at the same time the different covariates examined, the type of statistical analysis performed and the possible factors that could affect this relationship. The analysis of the main results of the literature search could provide new information on the possible interaction between pesticide use and autism spectrum disorder, which could help researchers set up new lines of research with the aim of deepening this potential association.

## 2. Methods

In this review, we explored the data that we found in the literature relating to the onset of ASD with early exposure to pesticides. The review was carried out according to the main items reported in the PRISMA checklist [19]. We searched on Web of Science and Google Scholar for original articles, published from 1977 to 2020 by using the following keywords: “pesticide” and “environmental pollutant”, combined with the keywords: “autism spectrum disorder, or ASD”, “pregnancy”, “early life” and “prenatal period”.

The literature search was conducted in both databases identifying the studies on the basis of their title and abstract and, subsequently, the entire articles were analyzed. The inclusion criteria for this study were the following:

- Studies involving humans;
- Scientific publications in English;
- Assessment of exposure to pesticides during pregnancy or in the first years of life;
- Case–control studies or cohort studies;
- Diagnoses of ASD in children based on the most recent diagnostic tools referring to DSM IV and V (Diagnostic and Statistical Manual of Mental Disorders, fourth edition and Diagnostic and Statistical Manual of Mental Disorders).

For each study involved in the review, we highlighted the following information:

- Year of publication and authors;
- Type of study;
- Size of the population sample taken into consideration;
- ASD outcome assessment;
- Exposure assessment;
- The different methods of analysis used;
- The adjustment variables taken into consideration;
- Exposure time window;
- Main results obtained.

We did not include studies carried out on animals or in vitro. Exploration of heterogeneity of the studies was performed by assessing their quality (i.e., level of evidence). Interpretation of the findings was conducted in the frame of current knowledge [20].

## 3. Results

**Figure 1:**
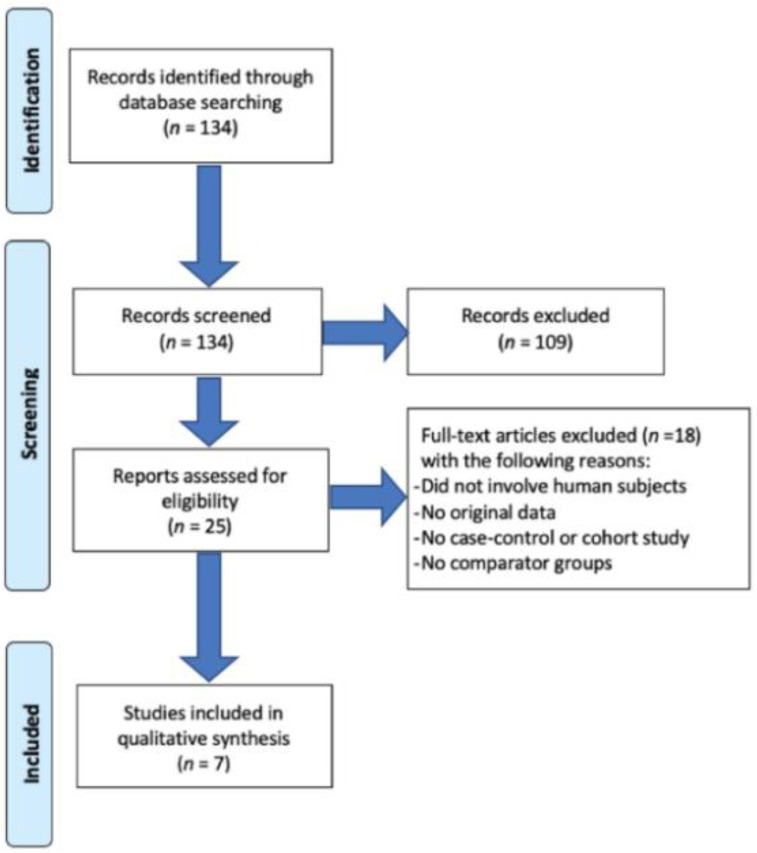
Flow diagram.

From a preliminary analysis of the available scientific literature, we selected a total of 134 references: twenty-five of them met the inclusion criteria based on title and abstract screening but only seven studies met the inclusion criteria after a full text screening and were selected for the review (Figure 1).

### 3.1. Association between Organophosphates and ASD

Organophosphates, currently among the most abundantly used insecticides in the world, are acutely toxic due to the inhibition of the enzyme acetylcholinesterase (AChE), which breaks down neurotransmitters at the post-synaptic membrane. However, neurodevelopmental and neurologic dysfunction occur at subacute levels, when AChE inhibition is absent [21]. However, these compounds also have other toxic mechanisms, including pro-inflammation, GABA signaling, thyroid disruption, oxidative stress induction and mitochondrial dysregulation, which are all potentially relevant for gestational exposures in early brain development [22,23]. In the literature we found several studies that examined the possible association between maternal exposure in pregnancy, and in the early years of the infant’s life, to organophosphate and a greater risk of the child being diagnosed with autism spectrum disorder. This association is supported by several case–control studies.

A Californian case–control study carried out in 2019, involving 2961 children diagnosed with autism spectrum disorder and controls in a 10:1 ratio, evaluated an association between the early exposure to pesticides and the onset of autism spectrum disorder. The results obtained showed a positive association between the exposure to organophosphate during pregnancy within 2000 m of the maternal residence and the risk of ASD onset. In particular, the risk of ASD was associated with prenatal exposure to glyphosate with an odds ratio (OR) of 1.16 (95% confidence interval (CI) 1.06, 1.27); chlorpyrifos with an OR of 1.13 (95% CI 1.05, 1.23); diazinon with an OR of of 1.11 (95% CI 1.01, 1.21); and malathion with an OR of 1.11 (95% CI 1.01, 1.22) [24].

Another Californian case–control study evaluated whether the residential proximity during pregnancy to areas where the pesticide organophosphate was sprayed was associated with the onset of ASD. A large weight of pounds of active ingredient applied for organophosphate, organochlorines, pyrethroids, and carbamates were aggregated within 1.25 km, 1.5 km, and 1.75 km buffer distances from the children’s homes. Gestation was associated with a 60% increased risk of ASD and became higher with exposures in the third trimester with an OR of 2.0 (95% CI 1.1, 3.6) or in the second trimester with an OR of 3.3 (95% CI 1.5, 7.4) [25].

Another case–control study that involved 296 cases from California and 220 controls recruited from the CHARGE study, examined the exposure to organochlorine pesticides combined with maternal folic acid (FA) intake and the onset of ASD. In this study population, the associations between pesticide exposure and ASD were attenuated by the maternal intake of high doses of FA (≥800 μg) during the first month of pregnancy compared to mothers with a low FA intake (<800 μg). In particular, the risk of ASD associated with prenatal exposure to organochlorine pesticides presented an OR of 0.8 (95% CI 0.5, 1.6) in mothers who took high amounts of FA in the first month of pregnancy, and an OR of 2.3 (95% CI 0.98, 5.3) in mothers who took low amounts of FA in the first month of pregnancy [26].

A 2018 cohort study involving participants from the CHAMACOS (Center for Health Assessment of Mothers and Children of Salinas), carried out in California, investigated the association between the prenatal exposure to organophosphate pesticides and typical traits related to the Autistic Spectrum. The exposure was assessed by measuring dialkyl phosphates (DAP) metabolites in urine, and the residential proximity to organophosphate pesticide (OP) use during pregnancy was assessed using California’s Pesticide Use Reporting (PUR) data; the estimated associations with ASD-related traits were assessed using linear regression models. The study found no clear evidence of an association between the residential proximity to OP use during pregnancy and ASD onset [25,27].

The main findings for the associations between OP and ASD are shown in Table 1.

### 3.2. Association between Organochlorines and ASD

Organochlorine pesticides (OCPs) are persistent organic pollutants (POPs), which include polychlorinated biphenyls, cyclodienes, hexachlorobenzene, dichlorodiphenyltrichloroethane and dichlorodiphenyl dichloroethylene. Their negative action is exerted through the persistent opening of the sodium channels and the interaction with the gamma-aminobutyric acid (GABA) receptors, with cause the interruption of the sodium/potassium currents of the nerve fibers [28,29]. Although their use has been banned in many countries, these compounds can still be detected in the environment and in animal and human tissues [30,31,32].

Many studies have investigated the possible association between maternal exposure in pregnancy and in the early years of the infant’s life to organochlorines and subsequent risk of developing autism spectrum disorder. This association is supported by two case–control studies.

In 2007, a case–control study evaluated whether gestational exposure to several common agricultural pesticides could induce autism spectrum disorder. In particular, the study evaluated the hypothesis that maternal residences close to the sites where agricultural pesticides are used during key periods of gestation could be associated with the development of ASD in children. The study involved 465 children with ASD who were born between 1996 and 1998 and up to 6975 controls. The results highlighted an association during the period immediately before and concurrent with the central nervous system embryogenesis (gestational weeks 1 through 8) by comparing the children of mothers living within 500 m of cultivated fields belonging to the highest non-zero quartile of organochlorine poundage, to children whose mothers were not living near to cultivated fields, showing an odds ratio for ASD of 6.1 (95% CI 2.4, 15.3). The ASD risk increased with the poundage of organochlorine applied and decreased with the distance from the fields [33].

A 2017 case–control study assessed whether the prenatal exposure to OCPs influenced children’s risk of ASD. The study was carried out in Southern California and involved 545 cases of ASD and 418 controls. The concentrations of OCPs measured in banked second-trimester serum samples were compared between the two groups, but no association with an increased risk of ASD was found [34]. The main findings for the associations between OCPs and ASD are shown in Table 2.

### 3.3. Association between Pyrethroids and ASD

Pyrethroid pesticides may directly activate microglial cells through their interaction with microglial VGSC (Voltage-Gated Sodium Channel). Because neuroinflammation plays a key role in many neurodegenerative diseases, these data provide an additional mechanism by which exposure to pyrethroid insecticides may contribute to neurodegeneration.

Pyrethroid insecticides cross the blood–brain barrier [35] and induce neurotoxicity by prolonging the opening of the VGSC [36]. Due to their potency, pyrethroids are widely used to control insect pests in agriculture, veterinary medicine, and domestic settings across the world [37].

The study of Shelton et al., already described above, also assessed the exposure of pregnant mothers to pyrethroid pesticide. The children of mothers living near to cultivated fields where pyrethroid is sprayed, just before conception or during the third trimester, were found to have an increased risk of both ASD and DD, with ORs ranging from 1.7 to 2.3 [25].

**Table 2:** Associations between organochlorine pesticides (OCPs) and ASD.

Authors and Year	Study Design	Sample Size	ASD Outcome Assessment	Exposure Assessment	Method of Analysis	Adjustment Variables	Time Window of Exposure	Main Findings
Roberts et al., 2007	Case-control	465 children born during 1996–1998 with ASD and 6975 controls.	Diagnosis of autism spectrum disorder based on the Diagnostic and Statistical Manual of Mental Disorders, fourth edition.	Proximity to pesticide applications was determined by California Department of Pesticide Regulation records refined using Department of Water Resources land use polygons.	Conditional logistic regressions	Maternal education and maternal race/ethnicity,	Pregnancy	Positive association during the period immediately before and concurrent with central nervous system embryogenesis (clinical weeks 1 through 8). Comparing children of mothers living within 500 m of cultivated fields with the highest non-zero quartile of organochlorine poundage, to those with mothers not living near cultivated fields suggested an odds ratio for ASD of 6.1 (95% confidence interval, 2.4–15.3).
Lyall et al., 2017	Case-control	545 ASD cases and 418 controls.	Diagnosis of autism spectrum disorder based on the Diagnostic and Statistical Manual of Mental Disorders, fourth edition.	Commercial pesticide application data from the California Pesticide Use Report (1997–2008) were linked to the addresses during pregnancy. Pounds of active ingredient applied for organophosphate, aggregated within 1.25 km, 1.5 km, and 1.75 km buffer distances from the homes.	Logistic regression	Child sex, month and year of birth), maternal age, maternal race/ethnicity, maternal weight at time of sample collection and parity.	Pregnancy	OCPs were not associated with increased risk of ASD,

The case–control study performed in California in 2017, which involved 296 cases and 220 controls recruited from the CHARGE study, also examined the exposure to pyrethroid pesticides combined with maternal folic acid intake and the onset of ASD. In particular, the risk of ASD associated with prenatal exposure to pyrethroid pesticides revealed an OR of 0.9 (95% CI 0.5, 1.8) in mothers who took high amounts of FA in the first month of pregnancy and an OR of 2.1 (95% CI 0.9, 4.8) in mothers who took low amounts of FA in the first month of pregnancy [26].

A 2019 Californian case–control study, involving 2961 children diagnosed with autism spectrum disorder and controls in a 10:1 ratio, evaluated an association between early exposure to pyrethroid pesticides and the onset of autism spectrum disorder. The obtained results showed a positive association between the exposure to pyrethroids during pregnancy, used within 2000 m from the maternal residence, and the risk of the onset of ASD. In particular, the risk of ASD was associated with the prenatal exposure to avermectin with an OR of 1.12 (95% CI 1.04, 1.22), and permethrin with an OR of 1.10 (95% CI 1.01, 1.20). The findings suggest that a child’s risk of autism spectrum disorder increases following prenatal exposure to ambient pesticides within 2000 m of their mother’s residence during pregnancy, compared with the children of women from the same agricultural region without such exposure [24].

Barkoski et al. assessed the relation between pyrethroid pregnancy exposure and autism spectrum disorder at 3 years. The participants were mother–child pairs (*n* = 177) in the MARBLES (Markers of Autism Risk in Babies Learning Early Signs) cohort that enrolled pregnant women who already had a child with ASD. The recurring maternal second and third trimester urine samples were analyzed for pyrethroid metabolite 3-phenoxybenzoic acid (3-PBA) showing that the prenatal 3-PBA concentrations were weakly associated with higher risk of ASD onset with an OR of 1.4 (95% CI 0.8, 1.6) [38]. The main findings for the associations between pyrethroids and ASD are shown in Table 3.

## 4. Discussion

This review summarizes seven cohort or case–control studies, involving children diagnosed with ASD according to DSM IV and V criteria, which examined the potential association between maternal exposure to pesticides, or exposure during the first years (from birth up to 3 years) of life, and an increased risk of developing ASD in children, grouping specific associations by pesticide (organophosphate, organochlorine, and pyrethroid). What emerges is an association between the maternal exposure to pyrethroid during pregnancy or in the early life of children and the risk of developing ASD. Three case–control studies showed a positive association between the exposure to pyrethroids during pregnancy or early life and ASD [24,25,26]. Moreover, a cohort study, highlighted an association between pyrethroid exposure during pregnancy or early life and ASD [38].

The results of this review, relating to the maternal exposure to organophosphate in pregnancy or in the first years of a child’s life and the risk of ASD onset, confirm the findings presented in a recent systematic review of human and preclinical models, which suggested that the gestational exposure to certain organophosphate agents could be linked to the clinical signs of ASD [18].

In our review, three case–control studies found a positive association between ASD and maternal exposure to organophosphate during pregnancy [24,25,26]. However, this type of association was not confirmed in the Sagiv et al. cohort study [27]. Indeed, there is limited evidence concerning the organochlorine pesticide and ASD, due to the small number of studies conducted and the low current use of these kinds of pesticides. Only two studies investigated this association: a case–control study found a positive association between organochlorine pesticides and ASD [33] while another case–control study found that OCPs were not associated with an increased risk of ASD [34].

The evidence indicating that the maternal exposure to pyrethroids may increase the risk of ASD is substantial, but this type of association is weak or limited with respect to the effect of organophosphate and organochlorine.

The number of articles included in our review is small because we have specifically chosen to consider only the cohort and case–control studies involving humans and those which take into account the diagnoses of ASD in children based on the most recent diagnostic tools: DSM IV and V (Diagnostic and Statistical Manual of Mental Disorders, fourth edition and Diagnostic and Statistical Manual of Mental Disorders). On the contrary, other reviews that included a higher number of papers also considered in vitro and animal studies [18]. Increasing the number of studies included in a future meta-analysis could help to improve the statistical power and highlight the possible associations between the onset of ASD and the exposure to atmospheric pollutants.

Furthermore, another important aspect that could be clarified concerns the understanding of the possible contribution of these pollutants taken as mixtures of pollutants and considered individually.

From the analysis of the involved studies, another aspect that emerges in this review is the critical time period, pregnancy and the first years of life, in which exposure to environmental pollutants seems to have the highest impact. In fact, symptoms related to autism spectrum disorder are commonly diagnosed during the first years of life (when the newborn should normally begin to interact with the world and society), thus suggesting a possible origin in the prenatal and early postnatal period, when neural connections occur at an astonishing rate [39]. Although the results obtained from the analysis of this review seem to confirm that pesticides may play an important role in the etiology of ASD, they represent only a small part of the environmental research on the causative agents involved in the development of ASD.

This type of analysis should consider different types of exposure, such as contaminants in water, food and soil. In fact, the studies we included in our review take into account other variables that could affect health, such as nutrition, smoking habits, social and educational aspects, the workplace, family structures, the neighborhood, and microbiome attributes.

From an epigenetic perspective, these exposures, unlike the inherited nuclear DNA, share the possibility of being modified, where this epigenome malleability opens the door to preventive interventions at various levels. Identifying and reducing exposure to these risk factors is the basis of the environmental-related research on autism [14].

The amount of evidence linking the exposure to pesticides and the occurrence of neurodevelopmental disorders, such as attention deficit hyperactivity disorder (ADHD) and learning difficulties, has increased dramatically over the years [40]. The exposure to chemicals and air pollutants begins during pregnancy and continues throughout life, causing the possible onset of adverse health outcomes in childhood or cognitive decline in the elderly [41].

Environmental factors can also affect the placenta, another key organ involved in the various processes that regulate fetal development. In fact, the placenta can be considered as an intermediate organ which, in the case of prenatal exposure to various pollutants, has the potential to express abnormal biological signatures that may prove to be useful as early indicators of the development of the disease later in life [42]. From the comparison of the selected studies, conflicting results emerge and this may be partly due to the different sizes of the population samples examined, the different statistical methods used to analyze the data and the different methods of diagnosing ASD among the several studies. On the contrary, the case–control studies showed more robust results and this could depend on the different confounding factors examined and the different methods of assessing the exposure to pesticides that may be a potential bias in case–control studies.

One aspect that could lead to confusion is linked to behavioral and lifestyle factors, such as smoking during pregnancy. Several studies have shown the direct association that this factor can have with a greater risk of developing ASD [43,44].

Some critical points of the studies we included in our analysis were: the limited comparisons between different geographical areas with different levels of pollution, the recruitment of small cohorts, the problem of quantifying and evaluating exposure not only for single molecules but also for their mixtures, the use of various models that allow the assessment of exposure to pollutants and the failure to use a standardized statistical method.

Therefore, future studies need to consider samples to be compared in different areas, and ensure the exposure of participants to a wide range of pollutant concentrations by measuring the individual exposure to different pollutants, considering various pathways (water, diet, air) combined and supplemented with genetic information [45,46].

Another aspect that should be considered for further studies, in order to quantify the individual exposure to a specific environmental pollutant, is the molecular alterations that cause changes in gene expression without modifying the DNA sequence, called epigenetic biomarkers [47]. These alterations, ranging from DNA methylation, histone modifications and microRNA (miRNA) expression, operate synergistically, affecting the genome-wide expression patterns in response to external stimuli [48,49].

Biomarkers that take into account specific exposures are able to measure the real internal doses caused by the environmental exposure pathways such as miRNAs. Small non-coding RNAs, involved in the regulation of gene expression at the post-transcriptional level, have proven to be potential biomarkers of environmental exposure, and thus are studied as biomarkers in various diseases [50,51]. Some studies have suggested that genetic factors, through epigenetic mechanisms, can interact with environmental factors, thus increasing the risk of ASD onset [10,52,53,54,55]. The potential association between genetics and the environment could be explained by epigenetic factors such as miRNAs [52].

## 5. Conclusions and Future Perspectives

Pesticides should be considered among the emerging risk factors for ASD, as there is evidence of the statistical association in case of prenatal and early life exposure, at least for some substances.

The potential association identified between the exposure to pesticides and ASD in this review needs to be implemented and confirmed by further epidemiological studies based on the individual assessment of outdoor and indoor exposures (i.e., through exposure biomarkers), including multiple confounding factors, and by using statistical models that take into account both single and multiple pesticide residues.

These efforts become necessary to determine the degree of causal association between pesticides and the onset of ASD.

## Figures and Tables

**Table 1:** Associations between organophosphates (OPs) and autism spectrum disorder (ASD).

Authors and Year	Study Design	Sample Size	ASD Outcome Assessment	Exposure Assessment	Method of Analysis	Adjustment Variables	Time Window of Exposure	Main Findings
von Ehrenstein et al., 2019	Case–control	2961 ASD cases and 10:1 controls	Diagnosis of autism spectrum disorder based on the Diagnostic and Statistical Manual of Mental Disorders, fourth edition.	Data from California state mandated Pesticide Use Reporting were integrated into a GIS (Geographical Information System) tool to estimate prenatal and infant exposures to pesticides.	Multivariable logistic regression model	Sex, year of birth, maternal age, maternal race/ethnicity, education and nitrogen oxides.	Pregnancy	Association with prenatal exposures to glyphosate with an odds ratio (OR) of 1.16 (95% confidence interval 1.06, 1.27), chlorpyrifos with an OR of 1.13 (95% CI 1.05, 1.23), diazinon with an OR of 1.11 (95% CI 1.01, 1.21) and malathion with an OR of 1.11 (95% CI 1.01, 1.22).
Shelton et al., 2014	Case–control	486 ASD case and 316 controls	Diagnoses of ASD were confirmed combining the Autism Diagnostic Interview, Revised (ADI^®^-R) and the Autism Diagnostic Observation Scale (ADOS).	Commercial pesticide application data from the California Pesticide Use Report (1997–2008) were linked to the addresses during pregnancy.	Multinomial logistic regression	Paternal education home ownership (binary), maternal place of birth child race/ethnicity and maternal prenatal vitamin intake.	Each trimester pregnancy, pregnancy and preconception	Proximity to orgnanophosphates during gestation was associated with a 60% increased risk of ASD and became higher with third-trimester exposures with an OR of 2.0 (95% CI 1.1, 3.6) and second-trimester exposures with an OR of 3.3 (95% CI: 1.5, 7.4).
Schmidt et al., 2017	Case–control	296 ASD cases and 220 controls	Diagnoses of ASD were confirmed by using the Autism Diagnostic Interview, Revised (ADI^®^-R) and the Autism Diagnostic Observation Schedule–Generic (ADOS-G).	Maternal supplemental folic acid (FA) and household pesticide product use were retrospectively collected in telephone interviews from 2003–2011.	Logistic regression models	Intake of vitamins B_6_ and D in the first month of pregnancy, home ownership and child’s year of birth.	Pregnancy	In particular, the risk of ASD associated with prenatal exposure to organochlorine pesticides had an OR of 0.8 (95% CI 0.5, 1.6) in mothers who took high amounts of FA in the first month of pregnancy and an OR of 2.3 (95% CI 0.98, 5.3) in mothers who took low amounts of FA in the first month of pregnancy.
Sagiv et al., 2018	Cohort	600 children	Autistic-related traits assessed with Social Responsiveness Scale, Version 2 (SRS-2) and meeting Diagnostic and Statistical Manual of Mental Disorders −5 (DSM-5) criteria for ASD.	California’s Pesticide Use Reporting (PUR) data.	Linear regression models	Maternal age, education, country of birth, marital status, depression, child’s age at assessment, sex and quality of the home environment.	Pregnancy	No clear evidence of associations between residential proximity to OP use during pregnancy and ASD-related traits.

**Table 3:** Associations between pyrethroids and ASD.

Authors and Year	Study Design	Sample Size	ASD Outcome Assessment	Exposure Assessment	Method of Analysis	Adjustment Variables	Time Window of Exposure	Main Findings
Shelton et al., 2014	Case-control	486 ASD cases and 316 controls	Diagnoses of ASD were confirmed combining the Autism Diagnostic Interview, Revised (ADI^®^-R) and the Autism Diagnostic Observation Scale (ADOS).	Commercial pesticide application data from the California Pesticide Use Report (1997–2008) were linked to the addresses during pregnancy. Pounds of active ingredient applied for pyrethroid, were aggregated within 1.25 km, 1.5 km, and 1.75 km buffer distances from the home.	Multinomial logistic regression	Paternal education home ownership (binary), maternal place of birth, child’s race/ethnicity, maternal prenatal vitamin intake.	Each trimester pregnancy, pregnancy and preconception	Children of mothers residing near pyrethroid insecticide applications just before conception or during third trimester were at greater risk for both ASD and DD, with ORs ranging from 1.7 to 2.3.
Schmidt et al., 2017	Case-control	296 ASD cases and 220 controls	Diagnoses of ASD were confirmed by using the Autism Diagnostic Interview, Revised (ADI^®^-R) and the Autism Diagnostic Observation Schedule–Generic (ADOS-G).	Maternal supplemental FA and household pesticide product use were retrospectively collected in telephone interviews from 2003–2011. High vs. low daily FA intake was dichotomized at 800 μg (median). Mothers’ addresses were linked to a statewide database of commercial applications to estimate agricultural pesticide exposure.	Logistic regression models	Home ownership, child’s year of birth, maternal intake of vitamins B_6_ and D in the first month of pregnancy.	Pregnancy	In particular, the risk of ASD associated with prenatal exposure to pyrethroid pesticides had an OR of 0.9 (95% CI 0.5, 1.8) in mothers who took high amounts of FA in the first month of pregnancy and an OR of 2.1 (95% CI 0.9, 4.8) in mothers who took low amounts of FA in the first month of pregnancy.
von Ehrenstein et al., 2019	Case-control	2961 ASD cases and 10:1 controls	Diagnosis of autism spectrum disorder based on the Diagnostic and Statistical Manual of Mental Disorders, fourth edition.	Data from California state mandated Pesticide Use Reporting were integrated into a GIS tool to estimate prenatal and infant exposures to pesticides (measured as pounds of pesticides applied per acre/month within 2000 m from the maternal residence).	Multivariable logistic regression model	Sex, year of birth, maternal age, maternal race/ethnicity, education, and nitrogen oxides.	Pregnancy	In particular, the risk of ASD was associated with prenatal exposure to avermectin with an OR of 1.12 (95% CI 1.04, 1.22) and permethrin with an OR of 1.10 (95% CI 1.01, 1.20).
Barkoski et al., 2018	cohort	177 mother-child pairs in the MARBLES cohort.	ASD cases defined by a child scoring at or above the ASD cutoff on the Autism Diagnostic Observation Scale (ADOS), the gold-standard diagnostic tool for ASD and meeting Diagnostic and Statistical Manual of Mental Disorders−5 (DSM-5) criteria for ASD.	Children were clinically assessed at 3 years and maternal second and third trimester urine samples were analyzed for pyrethroid metabolite 3-phenoxybenzoic acid (3-PBA).	Weighted multinomial logistic regression	Specific gravity, education, race/ethnicity, season and birth year.	Pregnancy	Prenatal 3-PBA concentrations were weakly associated with higher risk of ASD (OR 1.4, 95% CI: 0.8, 1.6).

## Data Availability

The data that support the findings of this study are available from the corresponding author, upon reasonable request.
